# Are acute:chronic workload ratios of perceived exertion and running based variables sensible to detect variations between player positions over the season? A soccer team study

**DOI:** 10.1186/s13102-022-00445-x

**Published:** 2022-03-28

**Authors:** Hadi Nobari, Ersan Arslan, Alexandre Duarte Martins, Rafael Oliveira

**Affiliations:** 1 Sports Scientist, Sepahan Football Club, Isfahan, Iran; 2 Department of Exercise Physiology, Faculty of Educational Sciences and Psychology, University of Mohaghegh Ardabili, Ardabil, Iran; 3 Department of Physiology, School of Sport Sciences, University of Extremadura, 10003 Cáceres, Spain; 4 Faculty of Sport Sciences, Tokat Gaziosmanpasa University, 60250 Tokat, Turkey; 5 Sports Science School of Rio Maior–Polytechnic Institute of Santarém, 2040-413 Rio Maior, Portugal; 6 Comprehensive Health Research Centre (CHRC), Departamento de Desporto E Saúde, Escola de Saúde E Desenvolvimento Humano, Universidade de Évora, Largo Dos Colegiais, 7000 Évora, Portugal; 7 Research Centre in Sport Sciences, Health Sciences and Human Development, 5001-801 Vila Real, Portugal; 8 Life Quality Research Centre, 2040-413 Rio Maior, Portugal

**Keywords:** Internal load, External load, High-speed running, Defenders, Midfielders, Strikers

## Abstract

**Background:**

The aim of this study was to describe and compare the in-season variations of acute: chronic workload ratio (ACWR) coupled, uncoupled, and exponentially weighted moving average (EWMA) through session rating of perceived exertion (s-RPE), total distance (TD), high-speed running distance (HSRD) and sprint distance (SPRINT) in three different periods of an elite soccer season according to player positions.

**Methods:**

Twenty male elite players (age: 29.4 ± 4.4) from an Asian First League team were daily monitored for twenty consecutive weeks during the 2017–2018 in-season. Forty-seven trainings and twenty matches were monitored using global positioning system units (GPS) to collect TD, HSRD and SPRINT. Through the collection of s-RPE, TD, HSRD, and SPRINT by ACWR and EWMA were calculated for each training session.

**Results:**

The results revealed that according to different periods of the season, workload measures observed in mid-season were meaningfully higher compared with early-season (g = ranging from 0.53 to 4.98) except for EWMA_SPRINT_. In general, wingers and strikers tended to have greater scores in workload measures compared to the defenders and midfielders (g = ranging from 0.41 to 5.42).

**Conclusions:**

These findings may provide detailed information for coaches and sports scientists regarding the variations of acute and chronic workload ratio and external loading in-season and between player positions in an elite soccer team.

## Background

In professional soccer, training and match load quantification is a common practice of coaches and their staff [1]. The load quantification could be divided in two dimensions: internal and external. Internal dimension is related to the psychophysiological state of the body [e.g. rated perceived exertion (RPE) and/or heart rate] while external dimension quantifies to the training prescribed by the coach and their staff (e.g. running speed distances and/or accelerometry-based variables [2].

In this sense, some research showed that player positions have different roles and consequently different intensities in training sessions [3–5] and matches [5–7], although not all studies present significant differences [8]. Considering training, on one hand, Malone et al. [9] showed higher total distance (TD) covered by central midfielders than other positions. The same authors showed higher RPE for strikers when compared to the other positions [9]. On the other hand, Clemente et al., [4] found higher values for wide defenders and wide midfielders with respect to high-speed running distance (HSRD) and number of sprints when compared with the other positions.

Regarding matches, Batista et al. [5] showed that during training, wide defenders achieved 64% of the sprint speed in matches, while central defenders, central midfielders, and central forwards achieved in training 107%, 100%, and 107%, respectively, than in matches. Dalen et al. [7] showed that different speed thresholds, player load, accelerations and decelerations were significantly different according to player positions during matches. Even before, Di Salvo et al. [6] showed that midfielders covered a higher TD than defenders and forwards during matches. Training sessions and matches together have great impact on the load experienced across the season. For instance, a recent study found differences in training load measures between playing positions [1, 5].

Beyond playing positions, soccer is a complex activity where other aspects such as the week-to-week and intra-week variations could influence data interpretations [10]. To analyse such variations, there are several methods that could help coaches and practitioners to better manage and periodize load such as acute: chronic work-load ratio (ACWR), that could be divided in coupled or uncoupled versions [11] and exponentially weighted moving average (EWMA) [12]. The coupled version of ACWR shows a ratio between the acute load of the last week with the chronic load of the last 28 days [13, 14] while the uncoupled version ACWR does not consider the most recent week for the chronic load [11]. Finally, EWMA provides specific ponderations through the different acute and chronic loads across the different weeks [12].

When analysing such workload measures and player positions together, studies are scarce [4, 15–17]. Recently, it was found that defenders presented higher values of ACWR values than midfielders and strikers [17]. One also found that strikers covered higher running speed distances than the defenders and midfielders [15] while the other found higher values for central midfielders than the other positions [17]. Other study found that wide defenders and wide midfielders displayed higher acute load for high-speed running distance (HSRD) and number of sprints compared to the remaining positions [4]. In addition, it was found that midfielders presented the highest weekly acute load of high metabolic load distance, while central defenders had the lowest value [16].

Furthermore, and to the best knowledge of the authors, only two studies analyse ACWR ratios through internal load measures such as the session rated perceived exertion and through external load measures such as the HSRD (> 19 km/h^−1^) and total distance (TD) covered. However, without considering the use of uncoupled ACWR or EWMA workload indexes [17, 18] and player positions [17].

Due to the limited research, especially in elite soccer teams, more evidence is needed to understand the variations across the season and between player positions. Therefore, the aim of this study was to describe and compare the in-season variations of ACWR coupled, uncoupled, and EWMA through session rating of perceived exertion (s-RPE), TD, HSRD and sprint distance (SPRINT) across different periods of an elite soccer season (early-, mid-, and end-season) according to player positions.

## Methods

### Participants

Twenty elite players (age: 29.40 ± 4.35 years old; body mass: 75.00 ± 3.87 kg; height: 1.79 ± 0.05 m; body mass index: 23.38 ± 1.79 years) from an Asian First League team participated in this study. The participants were divided according their field position: defenders (DF, n = 5), midfielders (MF, n = 5), (WG, n = 5), and strikers (ST, n = 5).

To be included in the analysis, the following inclusion criteria was adopted based on previous studies: (i) players were part of the team from week 1 to week 20; and (ii) players were regular participation in 80% of weekly training sessions [19–21]. The players with with prolonged injury or a lack of participation in training for at least two consecutive weeks or the players that presented the initial physical fitness tests 2 standard deviations below the squad mean were not considered in the sample. Finally, goalkeepers were excluded from the study due to intensity differences in training and matches [19–25].

### Experimental design

The present study is a descriptive-longitudinal approach. Only data from regular training sessions was considered for analysis which means that data from resistance training, competitions, rehabilitation and/or recuperation sessions was excluded. All sessions were planned by the coach and staff, and the researchers only controlled standardized the first and final 30 min of the sessions (i.e., before and after each training session). The analysed period ranged from the early-season (October 30, 2017) and lasted until the end-season (March 18, 2018). The present in-season was organized into three periods: early-season (weeks 1–7); mid-season (weeks 8–13); and end-season (weeks 14–20).

The number of the weeks and training sessions, number of competitive matches and total training duration (in average and total values) for player positions are presented in Table 1.

**Table 1:** Description of the present study

Periods of the in-season	Early-season	Mid-season	End-season
Number of weeks	7	7	6
Training sessions (N)	15	14	18
Training duration, average minutes, DF	62.82	73.18	78.54
Training duration, average minutes, MF	63.11	72.20	79.18
Training duration, average minutes, WG	60.35	73.32	76.26
Training duration, average minutes, ST	61.14	74.93	79.92
Training duration, total minutes, DF	1031.40	1273.00	1915.40
Training duration, total minutes, MF	1049.00	1285.00	1964.00
Training duration, total minutes, WG	978.20	1290.40	1890.60
Training duration, total minutes, ST	1014.20	1258.20	1837.40
Number of matches (N)	7	8	5

DF, defenders; MF, midfielders; WG, wingers; ST, strikers;

### External load monitoring

During the in-season, all training and match sessions were monitored using GPS (GPSPORTS systems Pty Ltd, Model: SPI High-Performance Unit (HPU); Australian). We provided all procedures about this GPS in previous studies [19–21, 26–28]. In addition, this GPS presented high validity and reliability [29].

### Internal load monitoring

Players were daily monitored through the CR-10 Borg’s scale [30], adapted by Foster et al. [31]. This scale showed validity and reliability to quantify the session intensity [16].

Thirty minutes after each session, players individually provided their RPE value using a tablet to avoid non-valid scores. The RPE values provided were also multiplied by the training duration, to obtain the s-RPE [31, 32]. Previously, all players were familiarized with RPE scale.

### Calculations of training indexes

Through s-RPE, TD, HSRD and SPRINT, the following measures were calculated: (i) ACWR, using coupled formula: dividing the acute workload (i.e., the 1-week rolling workload data), by the chronic workload (i.e., the rolling 4-week average workload data [11]); (ii) ACWR using uncoupled formula: dividing the weekly acute workload (i.e., the accumulated daily loads during 1-week), by the weekly chronic load (i.e., average of the three preceding weeks); and (iii) exponentially weighted moving averages (EWMA) [12]. The EWMA for a given day was calculated as:

$$EWMA_{today} = Load_{today} \times \lambda_{a} + \left( {\left( {1 - \lambda_{a} } \right) \times EWMA_{yesterday} } \right)$$

where $\lambda_{a}$ is a value between 0 and 1 that represents the degree of decay, with higher values discounting older observations in the model at a faster rate. The $\lambda_{a}$ is calculated as:

$$\lambda_{a} = 2/\left( {N + 1} \right)$$

where N is the chosen time decay constant, typically 7 and 28 days for acute (‘fatigue’) and chronic (‘fitness’) loads, respectively [12, 33].

### Statistical analysis

Descriptive statistics were used to characterize the sample. Shapiro–Wilk was used to test normality of results. Results were presented as mean ± standard deviation (SD). All measures obtained a normal distribution (Shapiro–Wilk > 0.05), it was used a repeated measures ANOVA test and the Bonferroni post-hoc test to compare measures for periods of the in-season and groups. The results are significant for a *p* ≤ 0.05. Hedge´s g effect size (ES) was also calculated to determine the magnitude of pairwise comparisons. The following criteria was used: The Hopkins threshold was utilized as follows: *g* ≤ 0.2, trivial; 0.2 < *g* ≤ 0.6, small; 0.6 < *g* ≤ 1.2, moderate; 1.2 < *g* ≤ 2.0, large; 2.0 < *g* ≤ 4.0, very large; and *g* > 4.0, nearly perfect [34]. All data were analysed using IBM SPSS Statistics (version 22, IBM Corporation (SPSS Inc., Chicago, IL).

## Results

**Fig. 1:**
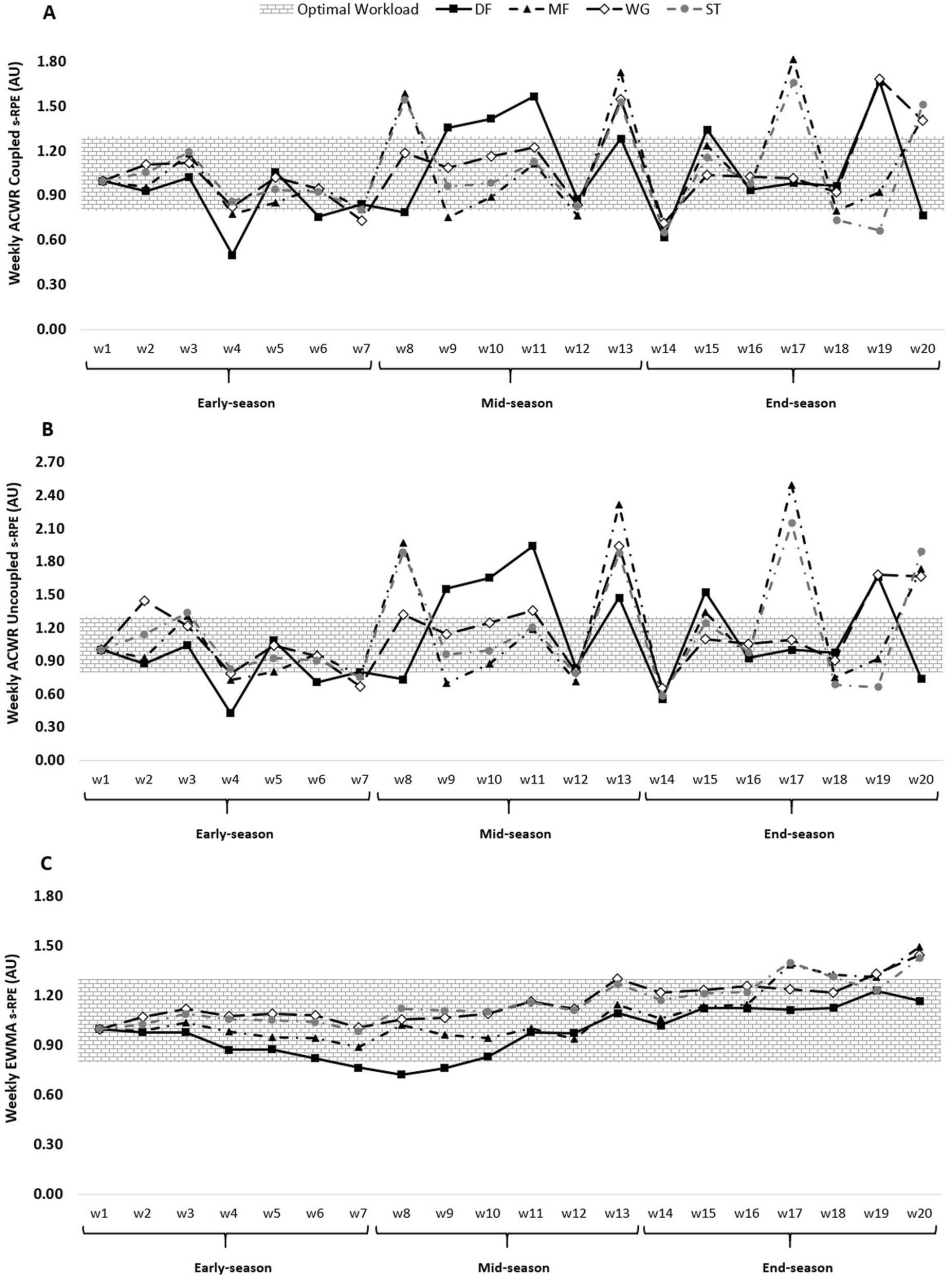
ACWR coupled (**A**) and ACWR uncoupled (**B**), and EWMA (**C**) variations calculated through the s-RPE across 20 weeks between players’ positions

**Fig. 2:**
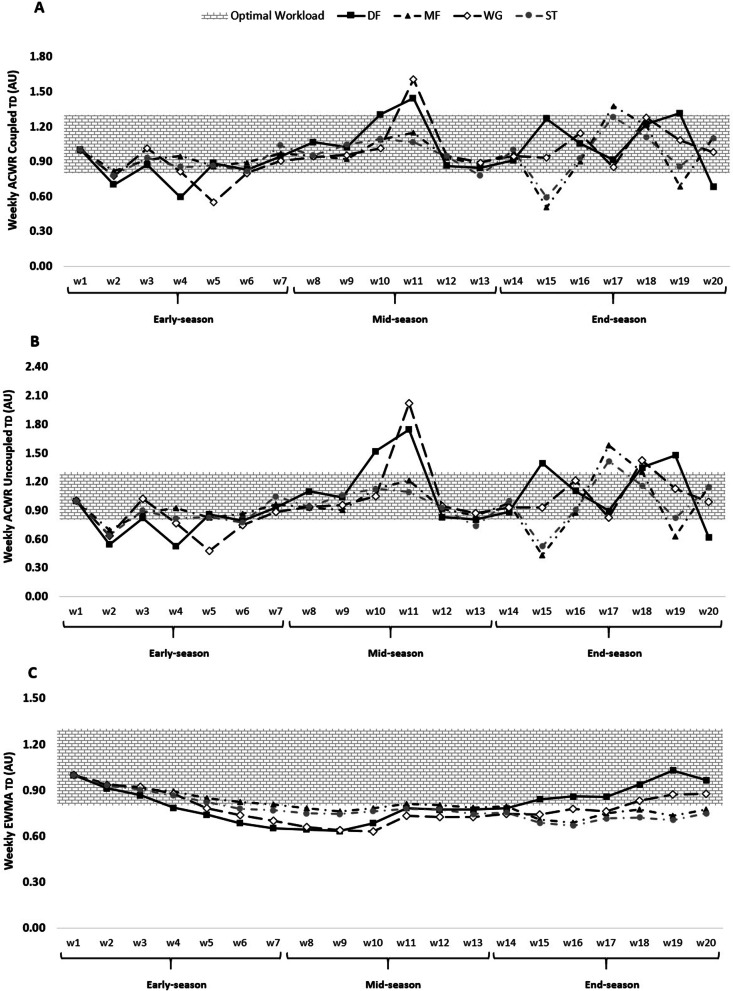
ACWR coupled (**A**) and ACWR uncoupled (**B**), and EWMA (**C**) variations calculated through the TD across 20 weeks between players’ positions

**Fig. 3:**
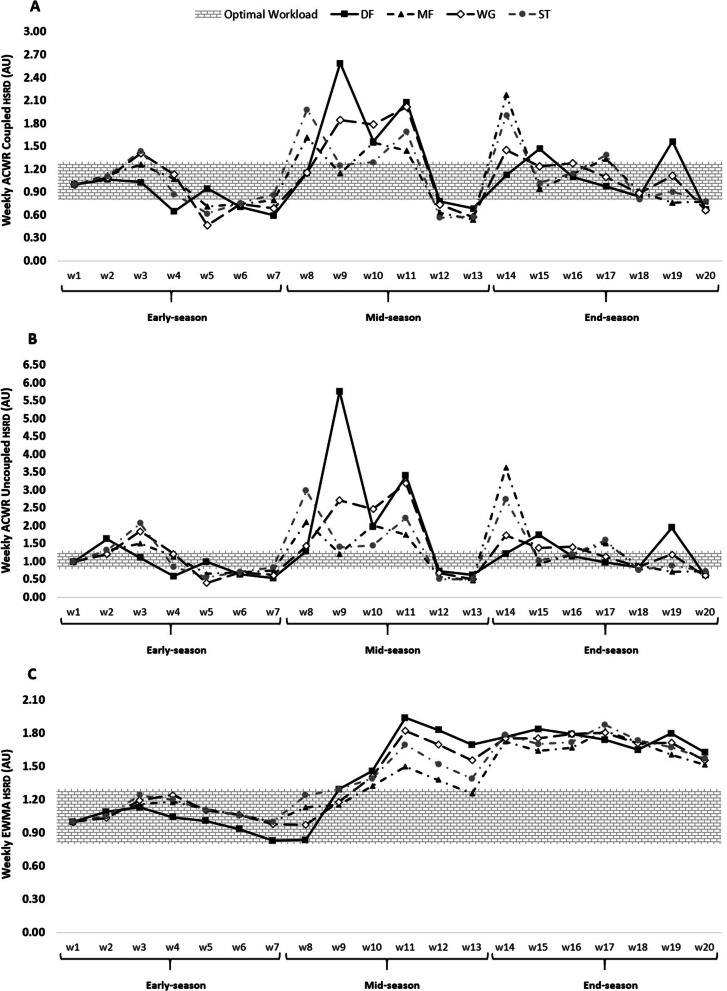
ACWR coupled (**A**) and ACWR uncoupled (**B**), and EWMA (**C**) variations calculated through the HSRD across 20 weeks between players’ positions

**Fig. 4:**
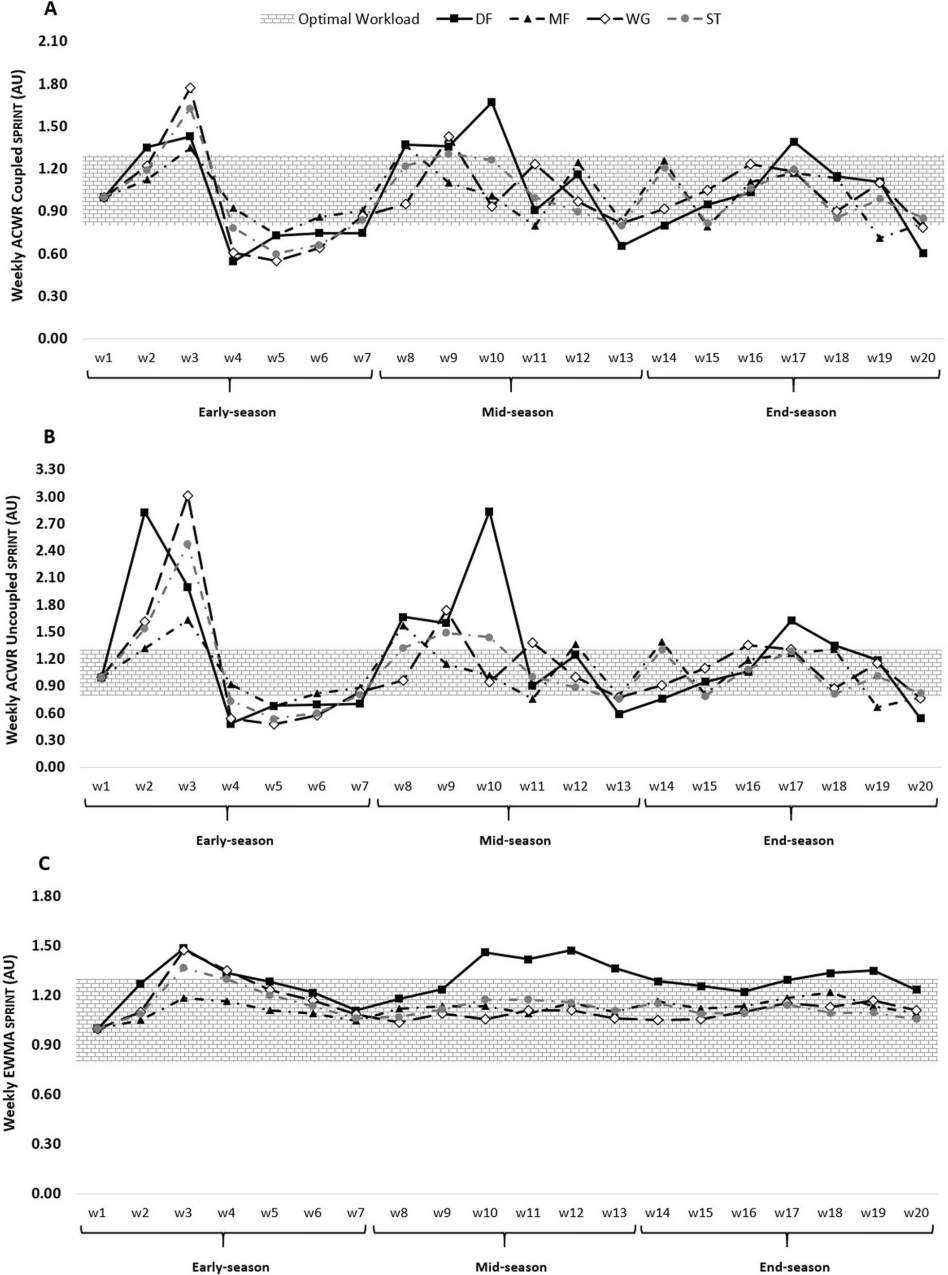
ACWR coupled (**A**) and ACWR uncoupled (**B**), and EWMA (**C**) variations calculated through the SPRINT across 20 weeks between players’ positions

Figures 1, 2, 3 and 4 show an overall view of the weekly average for ACWR coupled, ACWR uncoupled, and EWMA calculated through s-RPE, TD, HSRD, and SPRINT across different periods of an elite soccer season (early-, mid-, or end-season) between players’ positions.

The weekly changes in the aforementioned measures for s-RPE can be found in Fig. 1.

The weekly changes in the aforementioned measures for TD can be seen in Fig. 2.

The weekly changes in the aforementioned measures for HSRD can be seen in Fig. 3.

The weekly changes in the aforementioned measures for SPRINT can be found in Fig. 4.

Table 2 presents the differences between the early-season, mid-season, and end-season for ACWR coupled, ACWR uncoupled, and EWMA calculated through s-RPE, TD, HSRD, and SPRINT. To simplify the description, only large to nearly perfect effect sizes will be described here.

**Table 2:** Descriptive statistics (mean ± SD) of all measures in early-season, mid-season and end-season

Measures	EarlyS (Mean ± SD)	MidS (Mean ± SD)	EndS (Mean ± SD)	*p*	Hedges’ g (95% CI)
ACWR CP_s-RPE_ (AU)	0.94 ± 0.05	1.17 ± 0.04	0.93 ± 0.07	**EarS vs. MidS: < 0.01**	− 4.98 [− 6.37, − 3.78]^£^
				EarS vs. EndS: 0.687	–
				**MidS vs. EndS: < 0.01**	4.13 [3.07, 5.33]^£^
ACWR UCP_s-RPE_ (AU)	0.94 ± 0.09	1.31 ± 0.06	1.16 ± 0.12	**EarS vs. MidS: < 0.01**	− 4.74 [− 6.08, − 3.58]^£^
				**EarS vs. EndS: < 0.01**	− 2.03 [− 2.84, − 1.29]^§^
				**MidS vs. EndS: 0.001**	1.55 [0.86, 2.29]^#^
EWMA_s-RPE_ (AU)	0.99 ± 0.09	1.05 ± 0.13	1.24 ± 0.09	**EarS vs. MidS: 0.001**	− 0.53 [− 1.16, 0.09]^&^
				**EarS vs. EndS: < 0.01**	− 2.72 [− 3.65, − 1.89]^§^
				**MidS vs. EndS: < 0.01**	− 1.67 [− 2.42, − 0.96]^#^
ACWR CP_TD_ (AU)	0.87 ± 0.04	1.03 ± 0.06	0.88 ± 0.04	**EarS vs. MidS: < 0.01**	− 3.08 [− 4.07, − 2.19]^§^
				EarS vs. EndS: 0.534	*–*
				**MidS vs. EndS: < 0.01**	2.88 [2.03, 3.84]^§^
ACWR UCP_TD_ (AU)	0.82 ± 0.49	1.07 ± 0.11	1.04 ± 0.06	**EarS vs. MidS: < 0.01**	− 0.69 [− 1.34, − 0.06]*
				**EarS vs. EndS: < 0.01**	− 0.62 [− 1.26, 0.01]*
				MidS vs. EndS: 0.139	–
EWMA_TD_ (AU)	0.85 ± 0.03	0.74 ± 0.05	0.78 ± 0.08	**EarS vs. MidS: < 0.01**	2.61 [1.79, 3.52]^§^
				**EarS vs. EndS: 0.016**	1.14 [0.48, 1.83]*
				MidS vs. EndS: 0.065	–
ACWR CP_HSRD_ (AU)	0.93 ± 0.06	1.30 ± 0.13	0.98 ± 0.03	**EarS vs. MidS: < 0.01**	− 3.58 [− 4.67, − 2.62]^§^
				**EarS vs. EndS: < 0.01**	− 1.03 [− 1.71, − 0.38]*
				**MidS vs. EndS: < 0.01**	3.32 [2.40, 4.36]^§^
ACWR UCP_HSRD_ (AU)	0.99 ± 0.14	1.75 ± 0.41	1.27 ± 0.09	**EarS vs. MidS: < 0.01**	− 2.43 [− 3.30, − 1.64]^§^
				**EarS vs. EndS: < 0.01**	− 2.33 [− 3.19, − 1.55]^§^
				**MidS vs. EndS: < 0.01**	1.59 [0.89, 2.33]^#^
EWMA_HSRD_ (AU)	1.07 ± 0.14	1.42 ± 0.17	1.72 ± 0.08	**EarS vs. MidS: < 0.01**	− 2.20 [− 3.04, − 1.44]^§^
				**EarS vs. EndS: < 0.01**	− 5.59 [− 7.11, − 4.27]^£^
				**MidS vs. EndS: < 0.01**	− 2.21 [− 3.05, − 1.45]^§^
ACWR CP_SPRINT_ (AU)	0.96 ± 0.05	1.09 ± 0.07	0.88 ± 0.04	**EarS vs. MidS: < 0.01**	− 2.09 [− 2.91, − 1.35]^§^
				**EarS vs. EndS: < 0.01**	1.73 [1.02, 2.49]^#^
				**MidS vs. EndS: < 0.01**	3.61 [2.64, 4.71]^§^
ACWR UCP_SPRINT_ (AU)	1.12 ± 0.18	1.22 ± 0.25	1.05 ± 0.08	EarS vs. MidS: 0.211	–
				EarS vs. EndS: 0.134	*–*
				**MidS vs. EndS: 0.018**	0.89 [0.26, 1.56]*
EWMA_SPRINT_ (AU)	1.18 ± 0.15	1.18 ± 0.15	1.16 ± 0.11	EarS vs. MidS: 0.904	*–*
				EarS vs. EndS: 0.613	–
				MidS vs. EndS: 0.638	–

Significant differences between periods are highlighted in bold (*p* ≤ 0.05)

AU, arbitrary units; EarlyS, early-season; MidS, mid-season; EndS, end-season; SD, standard deviation; ACWR, acute: chronic workload ratio; EWMA, exponentially weighted moving averages; CP, coupled; UCP, uncoupled; s-RPE, session rate of perceived exertion; TD, total distance; HSRD, high‐speed running distance

^&^small effect; *moderate effect; ^#^large effect; ^§^very large effect; ^£^nearly perfect effect

The ACWR coupled_s-RPE_ shows a significant higher value in mid-season than early-season [nearly perfect effect] and shows a significant higher value in mid-season than end-season [nearly perfect effect]. The ACWR uncoupled_s-RPE_ presents a significant higher value in mid-season than early-season [nearly perfect effect], shows a significant higher value in end-season than early-season [very large effect], and shows a significant higher value in mid-season than end-season [large effect]. The EWMA_s-RPE_ shows a significant higher value in end-season than early-season [very large effect] and shows a significant higher value in end-season than mid-season [large effect].

The ACWR coupled_TD_ shows a significant higher value in mid-season than early-season [very large effect] and shows a significant higher value in mid-season than end-season [very large effect]. The EWMA_TD_ shows a significant higher value in early-season than mid-season [very large effect].

The ACWR coupled_HSRD_ presents a significant higher value in mid-season than early-season [very large effect] and shows a significant higher value in mid-season than end-season [very large effect]. The ACWR uncoupled_HSRD_ shows a significant higher value in mid-season than early-season [very large effect] and shows a significant higher value in end-season than early-season [very large effect]. The EWMA_HSRD_ shows a significant higher value in mid-season than early-season [very large effect], presents a significant higher value in end-season than early-season [nearly perfect effect], and shows a significant higher value in end-season than mid-season [very large effect].

Finally, the ACWR coupled_SPRINT_ presents a significant higher value in mid-season than early-season [very large effect], shows a significant higher value in early-season than end-season [large effect], and presents a significant higher value in mid-season than end-season [very large effect].

Table 3 presents the differences between player positions for ACWR coupled, ACWR uncoupled, and EWMA calculated through s-RPE, TD, HSRD, and SPRINT during in-season. There were no meaningful differences for EWMA_TD_, EWMA_HSRD_ and ACWR coupled_SPRINT_. To simplify the description, only large to nearly perfect effect sizes will be described here.

**Table 3:** Descriptive statistics (mean ± SD) of all measures between players’ positions

Measures	DF (Mean ± SD)	MF (Mean ± SD)	WG (Mean ± SD)	ST (Mean ± SD)	*p*	Hedges’ g (95% CI)
ACWR CP_s-RPE_ (AU)	1.06 ± 0.02	1.06 ± 0.02	1.07 ± 0.03	1.02 ± 0.03	DF vs. MF: 1.000	–
					DF vs. WG: 1.000	–
					DF vs. ST: 0.099	–
					MF vs. WG: 1.000	–
					MF vs. ST: 0.099	–
					**WG vs. ST:0.023**	1.51 [0.15, 3.11]^#^
ACWR UCP_s-RPE_ (AU)	1.16 ± 0.03	1.14 ± 0.03	1.16 ± 0.05	1.08 ± 0.03	DF vs. MF: 1.000	–
					DF vs. WG: 1.000	–
					**DF vs. ST: 0.010**	2.41 [0.84, 4.41]^§^
					MF vs. WG: 1.000	–
					MF vs. ST: 0.085	–
					**WG vs. ST: 0.015**	1.75 [0.35, 3.46]^#^
EWMA_s-RPE_ (AU)	1.10 ± 0.71	1.14 ± 0.09	1.16 ± 0.12	0.96 ± 0.05	DF vs. MF: 1.000	–
					DF vs. WG: 1.000	–
					DF vs. ST: 0.116	–
					MF vs. WG: 1.000	–
					**MF vs. ST: 0.025**	2.23 [0.72, 4.15]^§^
					**WG vs. ST: 0.011**	1.97 [0.51, 3.76]^#^
ACWR CP_TD_ (AU)	0.95 ± 0.01	0.95 ± 0.01	0.98 ± 0.02	0.99 ± 0.01	DF vs. MF: 1.000	–
					DF vs. WG: 0.091	–
					**DF vs. ST: 0.003**	− 3.61 [− 6.25, − 1.68]^§^
					**MF vs. WG: 0.029**	− 1.71 [− 3.40, − 0.32]^#^
					**MF vs. ST: 0.001**	− 3.61 [− 6.25, − 1.68]^§^
					WG vs. ST: 0.732	–
ACWR UCP_TD_ (AU)	0.95 ± 0.12	0.95 ± 0.01	0.99 ± 0.03	1.01 ± 0.01	DF vs. MF: 1.000	–
					**DF vs. WG: 0.017**	− 0.41 [− 1.70, 0.82]^&^
					**DF vs. ST: < 0.01**	− 0.64 [− 1.97, 0.60]*
					**MF vs. WG: 0.011**	− 1.62 [− 3.26, − 0.24]^#^
					**MF vs. ST: < 0.010**	− 5.42 [− 9.11, − 2.84]^£^
					WG vs. ST: 0.243	–
EWMA_TD_ (AU)	0.80 ± 0.02	0.79 ± 0.01	0.78 ± 0.01	0.81 ± 0.22	DF vs. MF: 0.804	–
					DF vs. WG: 0.430	–
					DF vs. ST: 1.000	–
					MF vs. WG: 1.000	–
					MF vs. ST: 0.220	–
					WG vs. ST: 0.109	–
ACWR CP_HSRD_ (AU)	1.03 ± 0.01	1.05 ± 0.01	1.06 ± 0.03	1.08 ± 0.01	DF vs. MF: 1.000	–
					DF vs. WG: 0.134	–
					**DF vs. ST: 0.019**	− 4.52 [− 7.67, − 2.27]^£^
					MF vs. WG: 1.000	–
					MF vs. ST: 0.250	–
					WG vs. ST: 1.000	–
ACWR UCP_HSRD_ (AU)	1.24 ± 0.04	1.27 ± 0.05	1.32 ± 0.09	1.44 ± 0.12	DF vs. MF: 1.000	–
					DF vs. WG: 0.917	–
					**DF vs. ST: 0.010**	− 2.02 [− 3.84, − 0.56]^§^
					MF vs. WG: 1.000	–
					**MF vs. ST: 0.040**	− 1.67 [− 3.34, − 0.29]^#^
					WG vs. ST: 0.216	–
EWMA_HSRD_ (AU)	1.35 ± 0.05	1.41 ± 0.03	1.42 ± 0.15	1.42 ± 0.17	DF vs. MF: 1.000	–
					DF vs. WG: 1.000	–
					DF vs. ST: 1.000	–
					MF vs. WG: 1.000	–
					MF vs. ST: 1.000	–
					WG vs. ST: 1.000	–
ACWR CP_SPRINT_ (AU)	0.96 ± 0.08	0.97 ± 0.02	0.96 ± 0.02	0.97 ± 0.02	DF vs. MF: 1.000	–
					DF vs. WG: 1.000	–
					DF vs. ST: 0.066	–
					MF vs. WG: 1.000	–
					MF vs. ST: 0.341	–
					WG vs. ST: 0.153	–
ACWR UCP_SPRINT_ (AU)	1.06 ± 0.02	1.09 ± 0.02	1.12 ± 0.05	1.23 ± 0.10	DF vs. MF: 1.000	–
					DF vs. WG: 0.858	–
					**DF vs. ST: 0.002**	− 2.13 [− 3.99, − 0.64]^§^
					MF vs. WG: 1.000	–
					**MF vs. ST: 0.009**	− 1.75 [− 3.46, − 0.35]^#^
					**WG vs. ST: 0.038**	− 1.26 [− 2.77, 0.05]^#^
EWMA_SPRINT_ (AU)	1.10 ± 0.03	1.16 ± 0.04	1.13 ± 0.11	1.29 ± 0.15	DF vs. MF: 1.000	–
					DF vs. WG: 1.000	–
					**DF vs. ST: 0.040**	− 1.59 [− 3.22, − 0.22]^#^
					MF vs. WG: 1.000	
					MF vs. ST: 0.308	–
					WG vs. ST: 0.126	–

Significant differences between player positions are highlighted in bold (p ≤ 0.05)

AU, arbitrary units; DF, defenders; MF, midfielders; WG, wingers; ST, strikers; SD, standard deviation; ACWR, acute: chronic workload ratio; EWMA, exponentially weighted moving averages; CP, coupled; UCP, uncoupled; s-RPE, session rate of perceived exertion; TD, total distance; HSRD, high‐speed running distance

^&^Small effect; *moderate effect; ^#^large effect; ^§^very large effect; £nearly perfect effect

The ACWR coupled_s-RPE_ shows a significant higher value in WG than ST [large effect]. The ACWR uncoupled_s-RPE_ presents a significant higher value in DF than ST [very large effect] and shows a significant higher value in WG than ST [large effect]. The EWMA_s-RPE_ shows a significant higher value in MF than ST [very large effect], shows a significant higher value in end-season than mid-season [large effect], and presents a significant higher value in WG than ST [large effect].

The ACWR coupled_TD_ shows a significant higher value in ST than DF [very large effect], shows a significant higher value in WG than MF [large effect], and shows a significant higher value in ST than MF [very large effect]. The ACWR uncoupled_TD_ presents a significant higher value in WG than MF [large effect] and shows a significant higher value in ST than MF [nearly perfect effect].

The ACWR coupled_HSRD_ shows a significant higher value in ST than DF [very large effect]. The ACWR uncoupled_HSRD_ presents a significant higher value in ST than DF [very large effect] and shows a significant higher value in ST than MF [large effect].

The ACWR coupled_SPRINT_ presents a significant higher value in ST than DF [very large effect], shows a significant higher value in ST than MF [large effect], and shows a significant higher value in ST than WG [large effect]. Finally, EWMA_SPRINT_ shows a significant higher value in ST than DF [large effect].

## Discussion

The main purpose of the present study was to compare the acute:chronic workload ratio (ACWR), exponentially weighted moving average (EWMA) through session rating of perceived exertion (s-RPE), total distance (TD), high-speed running distance (HSRD) and sprint distance (SPRINT) in three different periods of an elite soccer season according to player positions. To the best of the author’s knowledge, this is the first study to describe and compare the in-season variations of workload, including ACWR coupled, ACWR uncoupled and EWMA, between player positions in an elite soccer team. The results showed some significant differences in variations of workload measures in three different periods of a season according to player positions. Recently, several studies have increasingly provided information about internal and external training load monitoring, by using the accelerometer and global positioning system monitors in elite team sports especially soccer [18, 35–37]. Therefore, these systems help coaches and sports scientists to prevent the risk of injury [38], to increase player and team performance [39], and to observe variations of workload distribution [40] according to player positions in professional soccer teams.

Regarding the variations of ACWR coupled, ACWR uncoupled, and EWMA calculated through s-RPE between player positions, there were significant differences in all variables. One of the major findings of the study was that the highest values for ACWR coupled_s-RPE_ (1.81 arbitrary units (AU)), ACWR uncoupled_s-RPE_ (2.49 AU) and EWMA_s-RPE_ (1.49 AU) were observed in end-season for the midfielders (MF) which was different from a recent study [17] that found higher values for central defenders although without considering the uncoupled ACWR and EWMA calculations. Furthermore, the lowest values for ACWR coupled_s-RPE_ (0.50 AU) and ACWR uncoupled_s-RPE_ (0.43 AU) were found in early-season for the defenders (DF), while the lowest score in EWMA_s-RPE_ (0.72 AU) was recorded in mid-season for the DF. These findings are in line with previous studies monitoring workload, supporting that overall ACWR in trainings should be maintained within the ‘*sweet spot’* zone of 0.8–1.3 to minimize injury risk for players during the pre-season and in-season periods [11, 17, 41]. Our results also contrast with some previous study results [15, 18, 35]. Uday et al. [15] showed that the DF had the lowest value in ACWR (1.09 AU) with professional players from a team in First Portuguese League. Such differences between findings might be explained by the tactic strategies of the teams, length of the season and calculation system of ACWR.

Quantifying training and match external loading variations week to week or throughout a season in professional soccer teams is as important as the determination of internal loading to reduce injury risk and to regulate the training sessions loading according to playing positions and seasons. In the present study, the external loading variations of the professional team (TD, HSRD and SPRINT) were monitored by GPS throughout a season. Another important finding of the study was that the highest values for ACWR coupled_TD_ (1.61 AU) and ACWR uncoupled_TD_ (2.01 AU) were observed in mid-season for the MF, while the highest value in EWMA_TD_ (1.03 AU) was recorded in end-season for the DF. A recent study found higher and lower values for strikers during the first part of the in-season, respectively [17]. This results are in line with earlier study [17], which showed a significant differences between playing positions in different periods of an elite soccer season. These important differences provide new empirical evidence for coaches and sports scientists about how to reorganize and regulate the mesocycles and microcycles according to player positions in an elite soccer team. Similarly, the highest values for ACWR coupled_SPRINT_ (1.77 AU) and ACWR uncoupled_SPRINT_ (3.01 AU) were observed in early-season for the MF, while the highest value in EWMA_SPRINT_ (1.49 AU) was recorded in early-season for the DF. Contrast to these results, the highest values for ACWR coupled_SPRINT_ (2.59 AU), ACWR uncoupled_SPRINT_ (5.77 AU) and EWMA_HSRD_ (1.94 AU) were observed in mid-season for the DF. Several studies have recently investigated the competitive soccer players’ external load variations (related with distance-based measures) throughout a season, by using the accelerometry-based devices [16, 17, 42]. While many studies found small differences in TD, HSRD and SPRINT between player positions (especially between MF and the others) according to seasons [15, 16, 37], our results demonstrated higher differences in distance-based measures between player positions. We think that 2 main potential factors, such as the number of field positions, and tactical viewpoint, may explain the differences between the findings of our study and those of previous studies. From a practical point of view, in modern team sports especially soccer, the MF act as a bridge between DF and FW to build team strategy [43]. Therefore, increased team interactions with more distance covered and more running-based activities at high-intensity may affect tactical organizations and team performance.

The present study has some limitations that need to be acknowledged. This study was conducted on an elite male soccer team and presents one of the typical limitations which is the relatively small sample size. Therefore, our study results may not generalize with playing in amateur level and female soccer players. Another limitation is the lack of potentially important time-motion characteristics such as decelerations and accelerations covered during training and match play across the different periods of the season. Finally, there were some possible contextual factors, such as the match result [23, 44, 45] and location [24, 45] that could influence the results and should be considered for future studies. The final limitation of this study was the lack of internal and external load monitoring in resistance training and competitions sessions which should be considered in future studies.

However, the main strength of this study is the information regarding the in-season variations across the season and between player positions in an elite soccer team, describing relevant data for sport scientists and coaches. Considering the present study results, future studies should aim to investigate the in-season variations of acute and chronic workload in different sex and competitive level soccer players with larger sample sizes or more teams. Due to the limited research, especially in elite soccer teams, more evidence is needed to understand the variations across the season and between player positions. We believe that coaches and sports scientists need detailed empirical information from the further studies to better develop training strategies to maximize performance of the players for competitions.

## Conclusion

In summary, this study presents detailed information about the variations of acute and chronic workload ratio and external loading according to periods of in-season and player positions in an elite soccer team. The results demonstrated that the variables’ values observed in mid-season were meaningfully higher compared with early-season except for EWMA_SPRINT_. Overall, the wingers and strikers tended to have greater scores in all measurements compared to the defenders and midfielders according to player positions. From a practical point of view, coaches and sports scientists should take into account training load and the management of load for their players to observe variations of the indexes, to prevent the potential risk of injury and to increase players and team performance according to player positions in their soccer teams across the season. Furthermore, these important results might be beneficial for coaches and sports scientists to constitute optimal periodization in professional soccer teams..

## Data Availability

The datasets generated during and analyzed during the current study are available from the corresponding author on reasonable request.

## Abbreviations

ACWR: Acute:chronic workload ratio
EWMA: Exponentially weighted moving average
s-RPE: Session rating of perceived exertion
TD: Total distance
HSRD: High-speed running distance
SPRINT: Sprint distance
GPS: Global positioning system units
DF: Defenders
MF: Midfielders
WG: Wingers
ST: Strikers
HPU: High-Performance Unit
ES: Effect size
SD: Standard deviation
AU: Arbitrary units
